# Supplementary material for: The Cytotoxicity of Carbon Nanotubes and Hydroxyapatite, and Graphene and Hydroxyapatite Nanocomposites against Breast Cancer Cells
Source: Nanomaterials (Basel). 2023 Jan 30;13(3):556. doi: 10.3390/nano13030556 (PMC9919526; doi:10.3390/nano13030556)
Supplement: Supplementary file 1 [file nanomaterials-13-00556-s001.zip › nanomaterials-2133082-supplementary.pdf]

nanomaterials

Supplementary Materials

The Cytotoxicity of Carbon Nanotubes and Hydroxyapatite, and Graphene and Hydroxyapatite Nanocomposites Against Breast Cancer Cells

Tristan Nguyen [1], Anuj Maniyar [1], Mrinmoy Sarkar [1], Tapasree Roy Sarkar [1,*] and Gururaj M. Neelgund [2,*]

[1] Department of Biology, Texas A&M University, College Station, TX 77843, USA
[2] Department of Chemistry, Prairie View A&M University, Prairie View, TX 77446, USA
* Correspondence: tsarkar@bio.tamu.edu (T.R.S.); gmneelgund@pvamu.edu (G.M.N.)

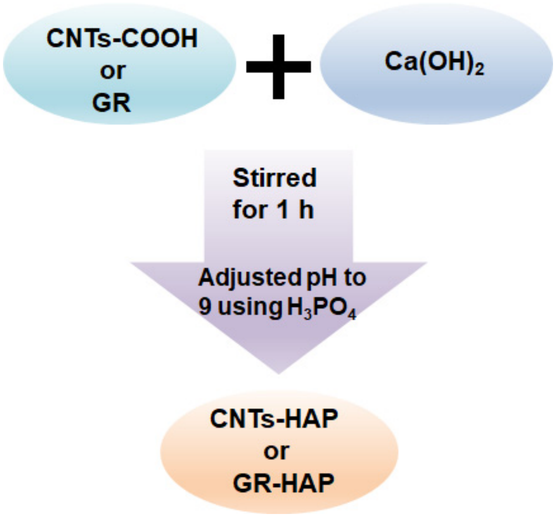

Scheme S1. Schematic representation of grafting of HAP over CNTs and GR.

Table S1. demonstrates the cytotoxicity of CNT-HAP on MCF7 cells.

	Control	5 µg/mL	25 µg/mL	50 µg/mL
**24 h**	100.00 ± 0.00	98.21 ± 2.83	67.91 ± 0.58***	24.51 ± 1.64***
**48 h**	100.00 ± 0.00	112.85 ± 29.44	59.58 ± 3.39***#	19.29 ± 0.49***#

***$p<0.001$ indicates significant difference compared to control, whereas #$p<0.05$ indicates significant difference compared to 24 h data.

Table S2. demonstrates the cytotoxicity of GR-HAP on MCF7 cells.

	Control	5 µg/mL	25 µg/mL	50 µg/mL
**24 h**	100.00 ± 0.00	106.96 ± 8.32	84.36 ± 4.70	44.77 ± 5.80***
**48 h**	100.00 ± 0.00	105.41 ± 6.88	66.10 ± 3.28***#	14.83 ± 2.57***##

***$p<0.001$ indicates significant difference compared to control, whereas #$p<0.05$ indicates significant difference compared to 24 h data.

Table S3. demonstrates the cytotoxicity of CNT-HAP on SUM-159 cells.

	Control	5 µg/mL	25 µg/mL	50 µg/mL
**24 h**	100.00 ± 0.00	85.93 ± 0.78***	45.53 ± 3.28***	17.90 ± 1.31***
**48 h**	100.00 ± 0.00	90.23 ± 3.91*	43.89 ± 2.38***	16.21 ± 2.78***

***$p<0.001$ and *$p<0.05$ indicates significant difference compared to control.

Table S4. demonstrates the cytotoxicity of GR-HAP on SUM-159 cells.

	Control	5 µg/mL	25 µg/mL	50 µg/mL
**24 h**	100.00± 0.00	88.06 ± 5.74**	41.16 ± 4.18***	30.46 ± 2.34***
**48 h**	100.00± 0.00	103.06 ± 8.45	56.98 ± 4.91***	22.77 ± 1.00***#

***$p<0.001$ and **$p<0.01$ indicates significant difference compared to control, whereas #$p<0.05$ indicates significant difference compared to 24 h data.
